# Temporal trend of gastric cancer burden along with its risk factors in China from 1990 to 2019, and projections until 2030: comparison with Japan, South Korea, and Mongolia

**DOI:** 10.1186/s40364-021-00340-6

**Published:** 2021-11-16

**Authors:** Xiaorong Yang, Tongchao Zhang, Hong Zhang, Shaowei Sang, Hui Chen, Xiuli Zuo

**Affiliations:** 1 Department of Gastroenterology, Qilu Hospital, School of Medicine, Cheeloo College of Medicine, Shandong University, 107 Wenhuaxi Road, Jinan, 250012 Shandong China; 2 Clinical Epidemiology Unit, Qilu Hospital of Shandong University, Jinan, China; 3 Laboratory of Translational Gastroenterology, Qilu Hospital of Shandong University, Jinan, China

**Keywords:** Gastric cancer, East Asia, Disease burden, Risk factors, Temporal trend, Projection

## Abstract

**Background:**

Identifying and projecting the epidemiological burden of gastric cancer (GC) can optimize the control strategies, especially in high-burden areas.

**Methods:**

We collected incidence, deaths, disability-adjusted life-years (DALYs), age-standardized incidence rate (ASIR), age-standardized mortality rate (ASMR), age-standardized DALY rate (ASDR) of GC from 1990 to 2019 in China, Japan, South Korea, and Mongolia from the Global Burden of Disease Study 2019. The average annual percentage change (AAPC) was calculated to quantify the temporal trends, and the projection was estimated by applying the Bayesian age-period-cohort model.

**Results:**

In China, the ASIR of GC declined slightly from 37.56/100000 in 1990 to 30.64/100000 in 2019 (AAPC of − 0.41), while the declines of ASMR and ASDR were pronounced (AAPC of − 1.68 and − 1.98, respectively), which were weaker than Japan and South Korea. Although the age-standardized rates of gastric cancer in most countries have declined overall in the past 30 years, the downward trend in the last 4 years has become flattened. Smoking remained one main contributor to DALYs of GC in China, Japan, South Korea, and Mongolia, with more than 24%. The contribution from high-sodium diet was similar between men and women, and kept relatively stable over the three decades. The predicted ASMRs among the four East Asian countries continued to decline until 2030, but the absolute deaths would still increase significantly, especially in South Korea and Mongolia.

**Conclusions:**

Although the age-standardized rates of GC in most countries have declined, the absolute burden of GC in the world, especially in China and Mongolia, is on the rise gradually. Low socio-demographic index and aging along with *Helicobacter pylori* infection, smoking, and high-salt diet were the main risk factors of GC occurrence and should be paid more attention.

**Supplementary Information:**

The online version contains supplementary material available at 10.1186/s40364-021-00340-6.

## Introduction

Gastric cancer (GC), a malignant tumor with the fifth most common cancer and the third leading cause of cancer death worldwide in 2020 [1]. The development of GC is caused by multiple environmental and genetic factors, including *Helicobacter pylori* (*H. pylori*) infection, unhealthy lifestyles, genetic predisposition, etc. [2, 3]. *H. pylori* infection is the well-known identified carcinogen for GC, infecting about 45% of the population worldwide [4], but only 1% of infection cases eventually develop GC [5]. Unhealthy lifestyles, such as tobacco smoking, alcohol drinking, dieting high in sodium, and intaking too little fresh vegetables or fruits, may increase the risk of GC [2]. Adopting several healthy lifestyle behaviors, including a healthy dietary pattern, smoking cessation, limiting alcohol consumption, and maintaining a normal body-mass index, has been reported to prevent 18.8% of all GC and 62.4% of cardia GC cases [6].

The epidemiological distribution of GC presents substantial geographical heterogeneity, and its highest age-standardized incidence rate (ASIR) occurred in the high-income Asia-Pacific region (29.5/100000) and East Asia (28.6/100000) in 2017, especially in Japan, South Korea, China and Mongolia [7]. Moreover, China accounted for nearly half of the global incident cases in 2017 [7]. Although China, Japan, South Korea, and Mongolia have many similarities in cultural backgrounds and East Asian lineage, the corresponding socio-economic development is at different levels. In the face of the great achievements made by South Korea and Japan in the prevention and treatment of GC [8, 9], China’s national cancer control plan was subsequently implemented [10, 11]. Comparing the disease burdens, temporal trends and risk factors of GC in these four East Asian countries will facilitate tracking the benefits of national screening and prevention programs.

The latest Global Burden of Diseases (GBD) Study 2019 provides a unique resource to grasp incidence, death, and disability-adjusted life-years (DALYs) of GC from 1990 to 2019 in 204 countries and territories [12], which expanded the coverage of the global GC burden in 185 countries by WHO/IARC. Complying with the latest GBD study, this present study aims to comprehensively compare the burden, trends and risk factors of GC burden from 1990 to 2019 by sex and age group in China, Japan, South Korea, and Mongolia, and further project the incidence and mortality burden of GC till 2030. Understanding the latest burden patterns will help policy-makers optimize the allocation of health resources to reduce the GC burden, especially in high-risk areas.

## Methods

### Data source

The exhaustive presentation of original data sources and processing methodology of the GBD 2019 study have been found in previous studies [12, 13]. The cancer burden information in the GBD study was estimated based on multiple national cancer registry systems and aggregate database of cancer registries, such as Cancer Incidence in Five Continents (CI5), Surveillance, Epidemiology, and End Results (SEER), and NORDCAN. Detailed information on original data sources used in the current study can be retrieved on the GBD 2019 Data Input Sources Tool website (http://ghdx.healthdata.org/gbd-2019/data-input-sources). The GC cases were identified based on the ICD9 and ICD10 codes pertaining to GC (151–151.9, 209.23, V10.04 and C16-C16.9, Z12.0, Z85.02-Z85.028, respectively) in the cancer systems. The detailed estimation flowchart and codes for cancer burden in the GBD study could be sought through the supporting address: http://ghdx.healthdata.org/gbd-2019/code/cod-2. In brief, the burden of GC in the GBD dataset was estimated by the following methods [7, 14, 15]: (1) Calculating the GC mortality-to-morbidity ratio (MIR) using all data sources covering both morbidity and mortality, and predicting the MIR for all locations using the Healthcare Access and Quality Index; (2) Collecting the incidence of GC from above-mentioned cancer registries; (3) Estimating GC mortality by multiply the cancer registration incidence data by the corresponding MIR; (4) Uploading all GC mortality information into the GC cause of death database, and using the Cause of Death Ensemble model (CODEm) process to assess the cancer-specific mortality of GC; (5) Further calculating the incidence of GC by the estimated cancer-specific mortality of GC and MIR.

In the GBD Study, the 95% uncertainty intervals (UIs) for all estimates were based on the 2.5th and 9.75th-ordered percentiles of 1000 random draws of the uncertainty distribution. We collected data on incidence, mortality, disability-adjusted life-years (DALYs), and its age-standardized rate (ASR) per 100,000 person-year of GC in China, Japan, South Korea, East Asia and Pacific region, and the world, covering the period of 1990–2019 from the GBD Study 2019 via the Global Health Data Exchange query tool (https://ghdx.healthdata.org/gbd-results-tool). The East Asia and Pacific region is defined by World Bank, including traditional East Asia, Southeast Asia, Australasia and Oceania (32 countries and territories) in our study. For fully describing the subnational disparity of GC burden in China, we analyzed data on a total of 34 province-level administrative units, including 22 provinces, five autonomous regions, four municipalities, Hong Kong Special Administrative Region (SAR), Macao SAR and Taiwan, which all were referred to as provinces throughout the subnational analysis. The subnational DALYs and age-standardized DALY rate of GC in 34 provinces in China in 2017 were retrieved from a previous study [16]. Expressed on a scale of 0 to 1, the socio-demographic index (SDI) is a comprehensive indicator reflecting the healthy development level, which is calculated based on income per capita, mean educational attainment, and fertility rates under 25 [12, 17].

### Risk factor analysis

The 87 risk factors in the GBD study were categorized into four major branches: environmental and occupational, behavioral, metabolic, and dietary risks [16]. The risk factors of each disease in the GBD study were identified by the GBD Collaborators based on the World Cancer Research Fund (WCRF) grades of convincing or probable evidence [16, 18]. Among them, smoking and high-sodium diet were judged to have adequate evidence to prove the causal relationship with GC development [7, 16], and the attributable proportions of risk factors for potential disease burden were estimated using the comparative risk assessment framework in GBD Study [7, 16]. Briefly, the framework of comparative risk assessment included the following steps: identifying forceful risk-outcome pairs, estimating relative risks, assessing the exposure levels and distributions, determining the theoretical minimum exposure level, calculating the population attributable fractions and attributable burden, and estimating attributable proportion for combined risk factors by considering the mediating effect.

### Statistical analyses

To avoid the effect of the age composition of the populations, the ASIR, age-standardized mortality rate (ASMR) and age-standardized DALY rate (ASDR) were used to estimate the difference among geographical regions, historical periods, and sexes, based on GBD world population age standard [19]. We conducted joinpoint trend analysis to observe the best-fit annual percentage changes (APC) in the ASR of GC from 1990 to 2019 in China, Japan, South Korea, Mongolia, East Asia and Pacific, and the world using Joinpoint Regression Program (version 4.8.0.1). Joinpoint trend analysis uses a piecewise linear regression to estimate the adaptive trend using one or more line segments [20]. Moreover, we calculated the average annual percentage change (AAPC) to describe the overall temporal trend in age-standardized rates of GC based on the following regression model, ln (ASR) = α + β* calendar year + ϵ, and the AAPC with its 95% confidence interval (CI) were derived from the formula of 100 × (exp (β) − 1) [19, 21, 22]. The Bayesian age-period-cohort (BAPC) model has been proven to have higher accuracy in predicting the cancer burden, especially in non-longer projection years, compared with the generalized additive model, smooth spline model, Nordpred model, Joinpoint model and Poisson regression [19, 23, 24]. In short, the age-period-cohort model is considered as a log-linear Poisson model assuming a multiplicative effect of age, period and cohort: *η*_*ij*_ *= log (λ*_*ij*_*) = μ + α*_*i*_ *+ β*_*j*_ *+ γ*_*k*_. In the model, *λ*_*ij*_ denotes the number of cases, *μ* is the intercept, and *α*_*i*_*, β*_*j*_ and *γ*_*k*_ are age, period, and cohort effects, respectively. *i* (*1 ≤ i ≤ I*) denotes age group at time *j (1 ≤ j ≤ J)*; *k* denotes cohort index, *k = M (I − i) + j*, which depends on age, period index, and the length of age group and period interval. *M* represents that the age group intervals are *M* times than the periodic intervals. In our study, 5-year age groups and yearly data were used, which implied that *M* was 5. In the current study, the BAPC model within the integrated nested Laplacian approximation (R packages BAPC and INLA) was used to project the burden of GC from 2020 to 2030, assuming the inverse gamma prior distribution of age, period, and cohort effects and applying second-order random walk (RW2) to adjust for excessive dispersion. The relationship between ASDR of GC and the SDI of 34 provinces of China was performed using Pearson correlation analysis. The main statistical analyses and graphing in the current study were implemented using R program version 4.0.3 (*https://www.R-project.org/*). Two-sided *P* value less than 0.05 was considered statistically significant.

## Results

### Comparison of GC burden in China, Japan, South Korea, Mongolia, East Asia and Pacific, and the world

**Fig. 1:**
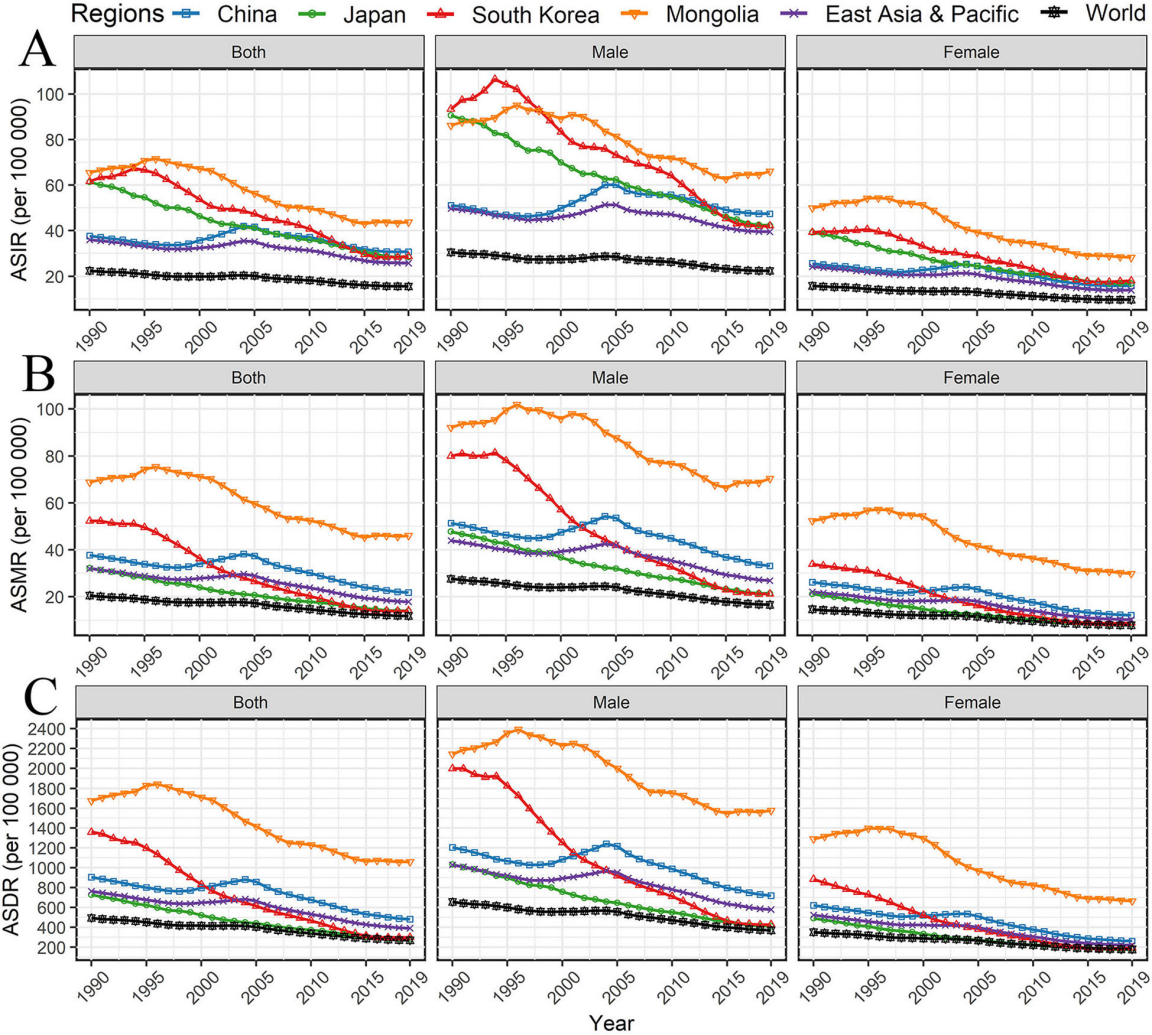
Trends for age-standardized rate of gastric cancer in China, Japan, South Korea, Mongolia, East Asia and Pacific, and the world from 1990 to 2019 by sexes. **A** ASIR/100000; **B** ASMR/100000; **C** ASDR/100000. ASIR age-standardized incidence rate, ASMR age-standardized mortality rate, ASDR age-standardized disability-adjusted life-year rate

Table 1 shows the and number and ASR of GC cases in China, Japan, South Korea, Mongolia, East Asia and Pacific, and the world from 1990 to 2019. China had 61.28*10^4^ incident cases of GC, with an ASIR of 30.6/100000 in 2019. In the same year, China had 30.55*10^4^ deaths due to GC, with an ASMR of 21.7/100000. Meanwhile, the DALYs were 98.25*10^4^, with an ASDR of 4.81/1000. In 2019, the incident cases, deaths, and DALYs of GC in China accounted for 76.74, 77.55, and 79.79% of GC burden in East Asia and Pacific, respectively, and 48.26, 44.03, and 44.21% of the global burden, respectively. The ASIR of GC in China declined slightly from 1990 to 2019 with AAPC of − 0.41(95%CI: − 0.77, − 0.06), conversely, the AAPC in ASMR and ASDR of GC in China were pronounced with − 1.68 and − 1.98. Notably, the GC burden in terms of incidence and mortality in Japan and South Korea ranked the top three in the world in 1990, but their ASRs were already lower than that of China in 2019 and their downward trends of GC burden was pronounced in the world, especially for South Korea (Fig. 1). The ASR of GC burden presented a decreasing trend across most countries in the world, but the ASR of GC burden in these four countries remained higher than the world, especially for ASIR (Fig. 1).

**Table 1:** The number, age-standardized rate, and changing trend of gastric cancer burden in China, Japan, South Korea, Mongolia, East Asia and Pacific, and the world from 1990 to 2019, by sexes

	Incidence					Mortality					DALYs				
	1990		2019		1990–2019	1990		2019		1990–2019	1990		2019		1990–2019
	No. *10^**4**^	ASR /10^**5**^	No. *10^**4**^	ASR /10^**5**^	AAPC (95% CI)	No. *10^**4**^	ASR /10^**5**^	No. *10^**4**^	ASR /10^**5**^	AAPC (95% CI)	No. *10^**4**^	ASR /10^**3**^	No. *10^**4**^	ASR /10^**3**^	AAPC (95% CI)
Both															
China	31.73	37.56	61.28	30.64	−0.41 (−0.77, − 0.06)	30.55	37.73	42.15	21.72	−1.68 (− 2.09, − 1.27)	82.49	9.06	98.25	4.81	−1.98 (− 2.4, − 1.57)
Japan	10.34	61.24	10.22	28.29	−2.73 (− 2.79, − 2.67)	5.33	32.19	5.72	14.07	−2.94 (− 2.99, − 2.89)	12.26	7.27	8.93	2.83	−3.36 (− 3.42, − 3.31)
South Korea	1.97	61.57	2.51	28.67	−3.24 (− 3.52, − 2.96)	1.59	52.35	1.22	14.09	−5.26 (− 5.50, − 5.03)	4.76	13.61	2.59	2.99	− 5.81 (−6.01, − 5.61)
Mongolia	0.07	65.41	0.10	43.70	−1.99 (−2.25,-1.74)	0.07	68.82	0.10	46.04	−1.98 (−2.24,-1.72)	1.84	16.75	2.77	10.59	−2.21 (− 2.48,-1.94)
East Asia & Pacific	47.99	35.95	79.85	25.77	−0.99 (−1.23, −0.75)	41.27	31.96	54.35	17.81	−1.89 (−2.19, − 1.59)	110.2	7.64	123.1	3.90	−2.20 (− 2.51, − 1.89)
World	88.34	22.44	126.98	15.59	−1.22 (− 1.36, − 1.08)	78.83	20.48	95.72	11.88	−1.87 (−2.04, − 1.71)	204.6	4.93	222.2	2.68	−2.11 (− 2.28, − 1.94)
Male															
China	20.75	51.07	45.13	47.35	0.14 (−0.21, 0.50)	19.71	51.36	29.85	33.14	−1.17 (−1.58, −0.77)	54.1	12.06	71.36	7.19	−1.47 (− 1.89, − 1.05)
Japan	6.67	90.61	6.72	42.16	−2.69 (−2.76, − 2.63)	3.34	47.75	3.61	21.31	−2.85 (− 2.91, − 2.80)	7.82	10.32	6.00	4.10	−3.27 (−3.32, −3.21)
South Korea	1.27	93.37	1.65	42.09	−3.32 (− 3.64, −2.99)	1.01	80.06	0.78	21.18	−5.27 (−5.51, −5.03)	3.04	19.99	1.74	4.28	−5.88 (−6.10, − 5.66)
Mongolia	0.04	86.18	0.06	66.04	−1.48 (−1.73,-1.23)	0.04	92.07	0.06	70.4	−1.5 (− 1.77,-1.23)	1.08	21.43	1.84	15.77	−1.62 (− 1.88,-1.37)
East Asia & Pacific	31.06	49.67	57.15	39.45	−0.56 (−0.80, −0.30)	26.32	43.98	37.46	26.83	−1.50 (− 1.81, − 1.20)	71.31	10.29	87.52	5.79	−1.78 (−2.11, − 1.45)
World	54.81	30.42	84.69	22.39	−0.96 (−1.12, − 0.81)	48.13	27.62	61.15	16.59	−1.70 (− 1.88, − 1.51)	127.9	6.55	145.2	3.69	−1.93 (−2.12, − 1.73)
Female															
China	10.98	25.57	16.15	15.80	−1.60 (− 1.95, − 1.25)	10.84	26.17	12.30	12.20	−2.59 (−3.00, − 2.17)	28.39	6.20	26.89	2.61	−3.02 (− 3.41, − 2.63)
Japan	3.67	39.14	3.51	16.86	−3.02 (− 3.10, −2.94)	1.99	21.19	2.11	8.39	−3.33 (− 3.41, − 3.24)	4.43	4.91	2.93	1.73	−3.75 (− 3.85, − 3.66)
South Korea	0.70	39.25	0.86	18.10	−3.35 (− 3.60, − 3.10)	0.58	33.88	0.44	8.95	−5.43 (−5.72, −5.15)	1.71	8.84	0.85	1.92	−5.87 (−6.09, − 5.65)
Mongolia	0.03	49.90	0.03	28.18	−2.62 (−2.90,-2.33)	0.03	52.34	0.03	29.86	−2.55 (− 2.84,-2.26)	0.76	12.90	0.93	6.65	−3.00 (−3.31,-2.69)
East Asia & Pacific	16.93	24.25	22.70	13.88	−1.94 (−2.17, −1.71)	14.96	22.02	16.89	10.3	−2.65 (− 2.94, − 2.35)	38.88	5.22	35.62	2.19	−3.08 (− 3.36, − 2.79)
World	33.53	15.81	42.29	9.71	−1.76 (− 1.88, − 1.64)	30.7	14.68	34.57	7.92	−2.22 (− 2.36, − 2.08)	76.73	3.51	77.00	1.78	−2.46 (− 2.59, − 2.33)

*DALYs* disability-adjusted life-years, *No*. number, *ASR* age-standardized rate, *AAPC* average annual percentage change, *CI* confidence interval

### Sex disparity of GC burden

Globally, 66.70% (84.69*10^4^ cases) of the incident cases of GC occurred in men, compared with 33.30% (42.29*10^4^ cases) in women in 2019 (Table 1). The global ASIR of GC in men was approximately 2.31 times higher than women in 2019, and the gender disparity further widened over the past 30 years because of the pronounced decline in women. Similarly, 63.88% (61.15*10^4^ deaths) of the global deaths occurred in men, compared with 36.12% (34.57*10^4^ deaths) in women. The ASMR in men was about 2.10 times higher than that in women.

In 2019, 73.65% (45.13*10^4^ cases) of the GC incident cases occurred in men, compared with 26.35% (16.15*10^4^ cases) in women in China, with a male-to-female ratio of 2.99 (Table 1). Among all GC deaths in China, males accounted for 70.82% (29.85*10^4^) and females accounted for 29.18% (12.30*10^4^), with a male-to-female ratio of 2.71 (Table 1). In contrast, gender disparities in ASIR and ASMR between the sexes were narrowed in 2019 for Japan, South Korea and Mongolia (ASIR in Japan: the male-to-female ratio was 2.50; ASMR in Japan: 2.54; ASIR in South Korea: 2.33; ASMR in South Korea: 2.38; ASIR in Mongolia: 2.34; ASMR in Mongolia: 2.35). Notably, the female ASIR of GC in China was always less than that in Japan, South Korea and Mongolia.

### Age composition of GC burden

**Fig. 2:**
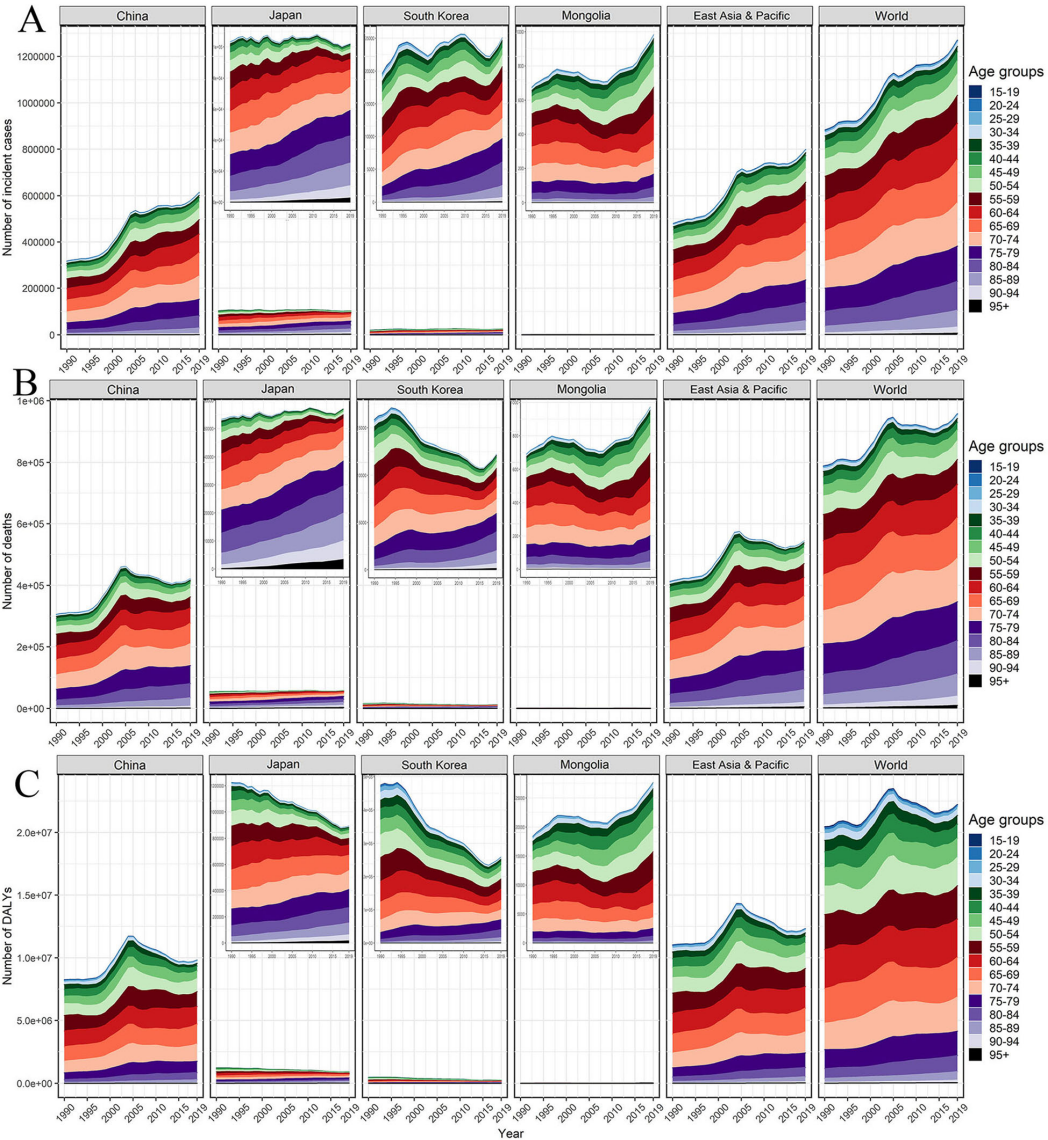
Trends for the age-specific absolute burden of gastric cancer in China, Japan, South Korea, Mongolia, East Asia and Pacific, and the world from 1990 to 2019, both. **A** Number of incident cases; **B** Number of deaths; **C** Number of DALYs. DALYs, disability-adjusted life-years

The ASR of GC burden usually increased sharply after 45 years of age and reached a peak at the age of 85 in the world, East Asia and Pacific, and the four countries, which was evident in males (Fig. S1). For the absolute GC burden, China accounted for a more significant proportion of the GC patients under 70 years, usually exceeding 50% in 2019 (Fig. S2). From 1990 to 2019, the proportion of elderly people over 70 among all GC patients continued to increase (Fig. 2). Except for the elderly over 90, the global incidence rate of GC dropped in all age groups over the past 30 years, especially those under 75 with AAPCs between − 2 and − 1 (Fig. S3). Similar age patterns could be observed in GC mortality and DALYs, and this is more pronounced in Japan and South Korea (Fig. S3-S5). Albeit the incidence of GC under 70 years in Japan and South Korea exceeded the average level of the world, the corresponding rates for mortality and DALYs in Japan and South Korea were closed to the world, or even lower (Fig. S3-S5).

### Changing trends and projection in GC burden

**Fig. 3:**
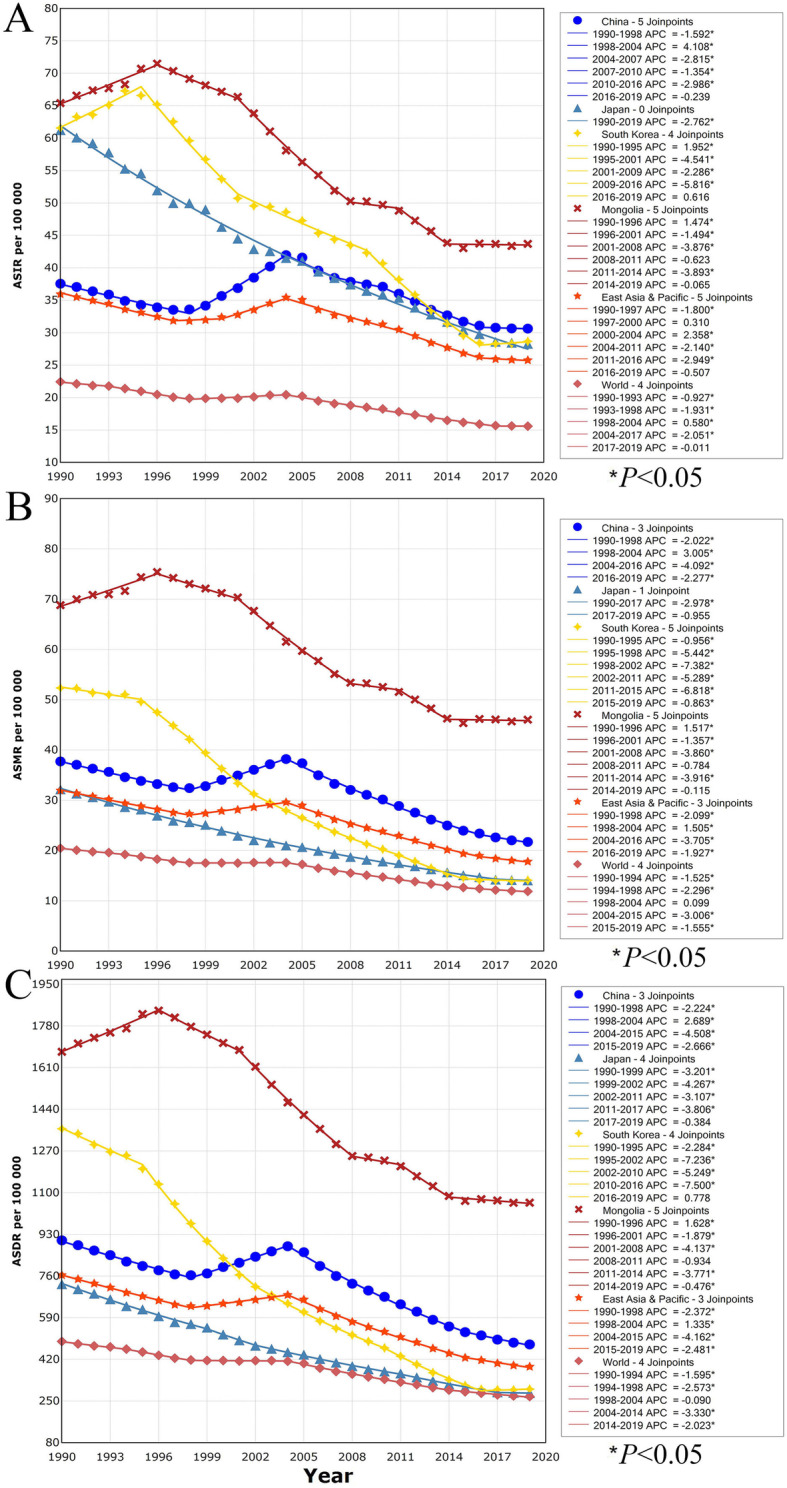
Trends for the ASR of gastric cancer in China, Japan, South Korea, Mongolia, East Asia and Pacific, and the world from 1990 to 2019 calculated by joinpoint regression analyses. **A** ASIR among both sexes; **B** ASMR among both sexes; **C** ASDR among both sexes. ASIR, age-standardized incidence rate; ASMR age-standardized mortality rate; ASDR, age-standardized disability-adjusted life-year rate

The results of the joinpoint regression analyses are shown in Fig. 3. In China, the ASIR of GC changed in six periods, and the APC was − 0.25 in the last period from 2016 to 2019 (Fig. 3A, *P* > 0.05). Similarly, the ASMR in China underwent six periods, but the ASMR was decreasing significantly since 2004 (Fig. 3B). The ASDR in China changed in four stages, and the APC was − 2.67 in the last stage from 2015 to 2019 (Fig. 3C, *P* < 0.05). Notably, the ASR of GC burden increased from 1998 to 2004. During the past three decades, Japan, South Korea and Mongolia showed different trend patterns in ASR of GC. Overall, the ASIR was declining significantly for nearly 20 years, but the descending trend somewhat plateaued in recent years (Fig. 3). Moreover, all ASRs of global GC burden declined underwent five stages. Although the APC of ASIR was − 0.01 in the last period from 2017 to 2019 (*P* > 0.05), the ASMR and ASDR of GC were declining since 2004 (Fig. 3).

**Fig. 4:**
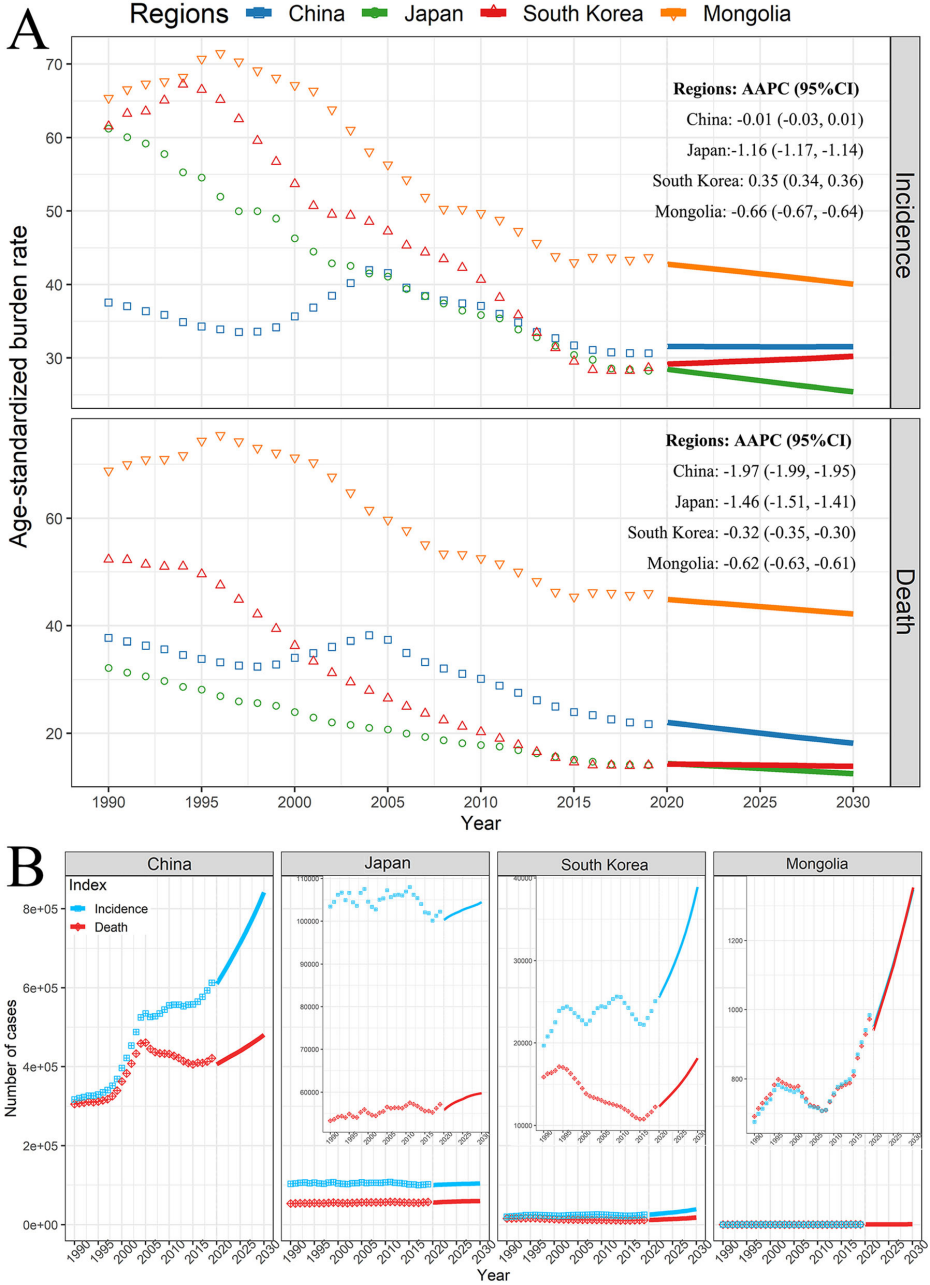
The projection of GC from 2020 to 2030 in China, Japan, South Korea, and Mongolia. **A** Age-standardized burden rate; **B** The absolute burden. The dots present the observed value. The solid lines present the predicted value. AAPC, average annual percentage change from 2020 to 2030; CI, confidence interval

Based on the GBD data of GC from 1990 to 2019 in China, Japan, South Korea, and Mongolia, the morbidity and mortality of GC in the next 11 years were estimated (Fig. 4). From 2020 to 2030, the ASIR of GC in Japan and Mongolia continued to decline from 28.0/100000 in 2020 to 24.9/100000 in 2030 with AAPC of − 1.16, and from 42.3/100000 in 2020 to 39.6/100000 in 2030 with AAPC of − 0.66, respectively, but China’s ASIR remained stable at around 31.0/100000 (AAPC = − 0.01), and South Korea’s ASIR even elevated slightly from 28.7/100000 in 2020 to 29.7/100000 in 2030 (AAPC = 0.35). As for ASMR of GC, these countries all showed a significant decline, with the AAPCs of China, Japan, South Korea, and Mongolia were − 1.97 (from 21.5/100000 in 2020 to 17.7/100000 in 2030), − 1.46 (from 13.9/100000 in 2020 to 12.0/100000 in 2030), − 0.32 (from 13.8/100000 in 2020 to 13.4/100000 in 2030), and − 0.62 (from 44.4/100000 in 2020 to 41.7/100000 in 2030) respectively (Fig. 4A). The absolute burden of incident cases and deaths in the four countries would be on the rise gradually, especially in South Korea and Mongolia with the growth exceeding 40% (Fig. 4B). In 2030, the projected incident cases of GC in China, Japan, South Korea, and Mongolia would be 841,494, 104,453, 38,915, and 1347 respectively, and the corresponding deaths would be 480,599, 59,756, 18,143 and 1353, respectively.

### GC burden in subnational areas in China

**Fig. 5:**
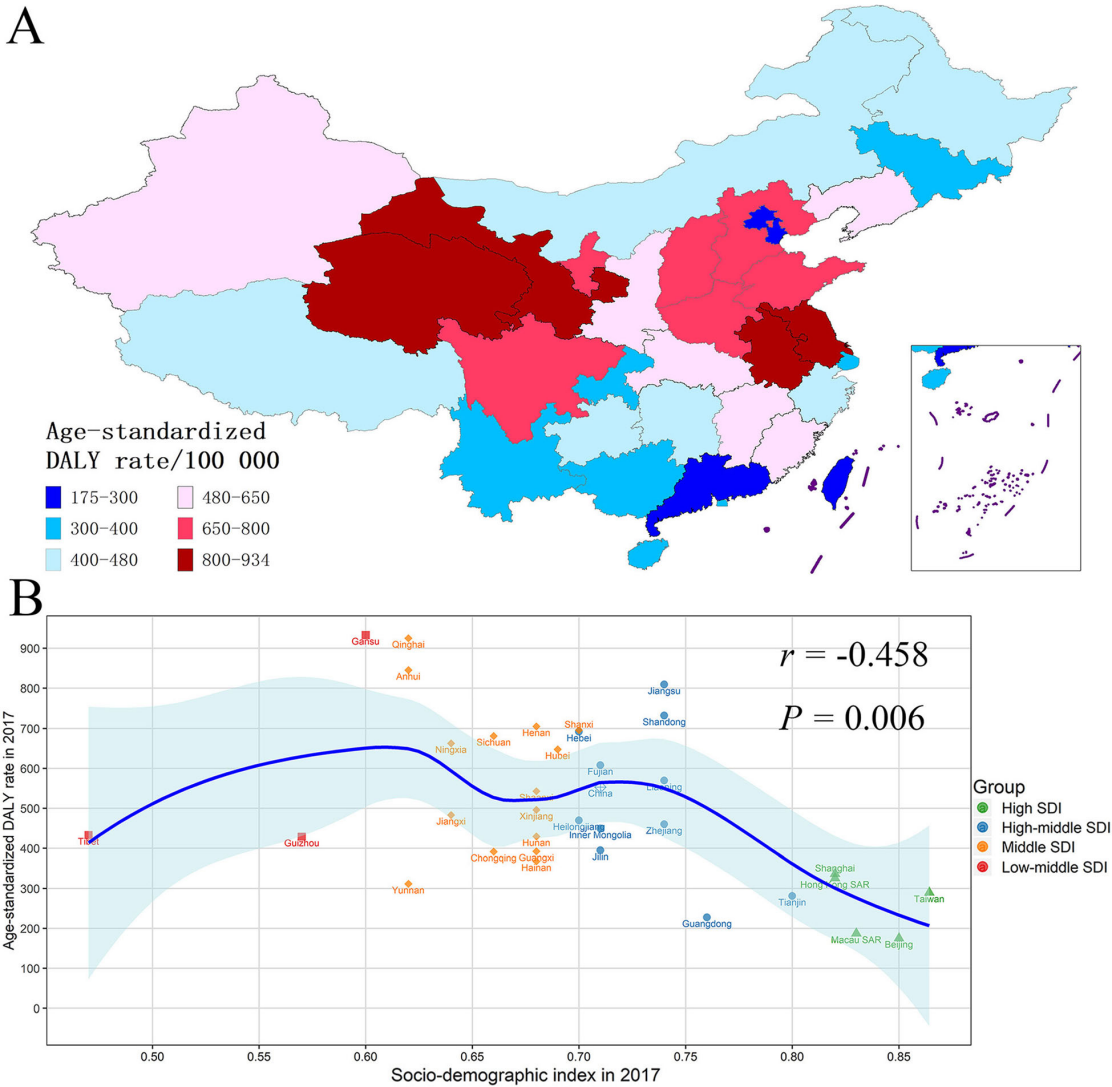
The subnational burden of gastric cancer in China. **A** The age-standardized DALY rate of gastric cancer in the 34 provinces of China in 2017. **B** Age-standardized DALY rate and socio-demographic index in the 34 provinces of China in 2017. DALY disability-adjusted life-year

The DALYs and ASDR of GC in the 34 provinces of China between 1990 and 2017 were found in Table S1. The variety of ASDR of GC was close to 5.34 times across the 34 provinces of China, and the four provinces with the greatest ASDR were Gansu (933.14/100000), Qinghai (925.18/100000), Anhui (845.54/100000), and Jiangsu (809.53/100000). In contrast, the lowest ASDR was in Beijing (175.50/100000) (Fig. 5A, Table S1). We additionally noted the ASDR of GC of Mongolia was 2.38 times that of Inner Mongolia. The relationship between ASDR of GC and SDI values in the 34 provinces of China in 2017 is presented in Fig. 5B, which showed the provinces with higher SDI usually had lower ASDR of GC (*r* = − 0.458, *P* = 0.006).

### Risk factors of GC in China, Japan, South Korea, Mongolia, East Asia and Pacific, and the world

**Fig. 6:**
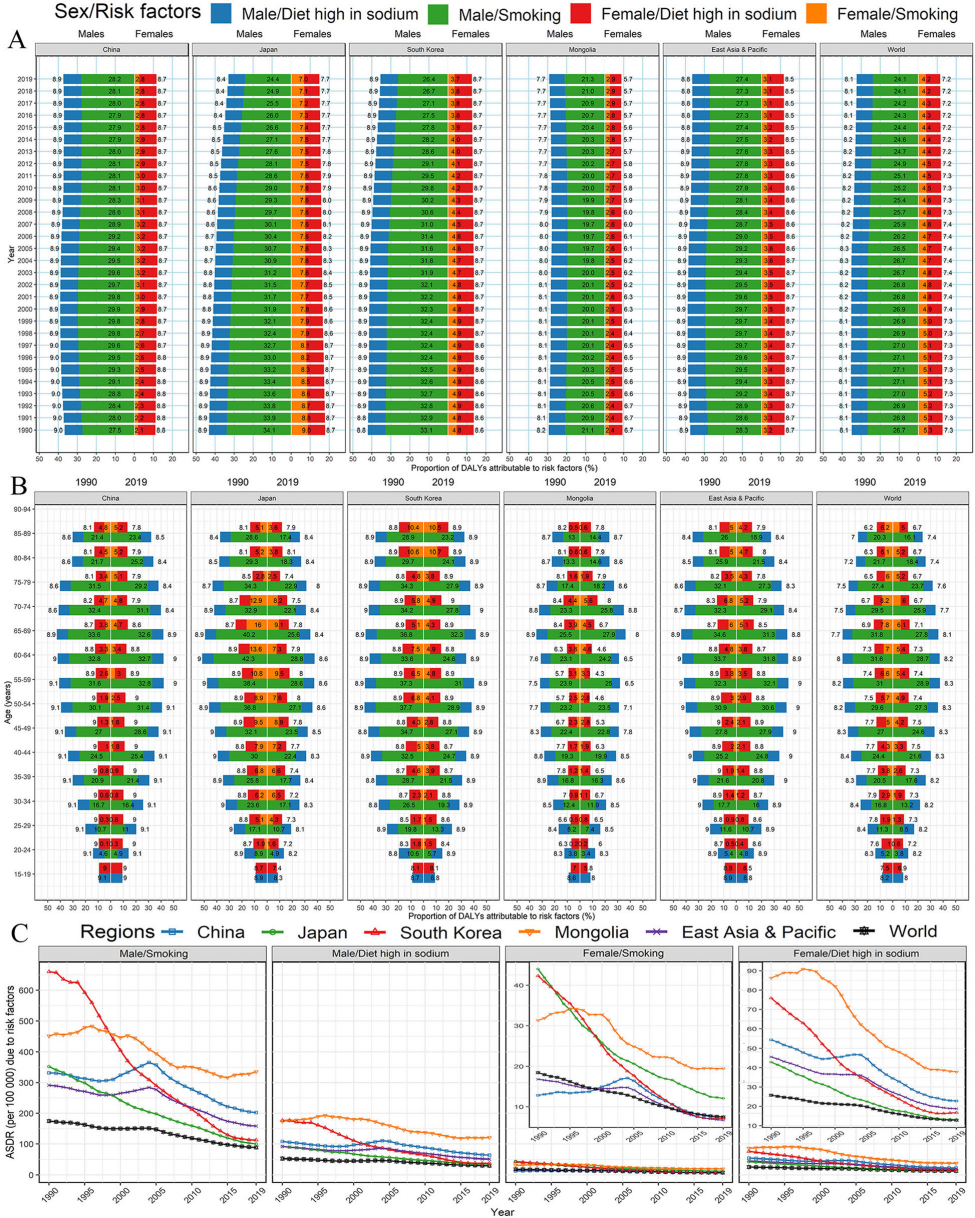
Percentage of gastric cancer DALYs attributable to dieting high in sodium and smoking in China, Japan, South Korea, Mongolia, East Asia and Pacific, and the world, by sexes. **A** Trends for the percentage of age-standardized DALY rate from 1990 to 2019. **B** Age-specific percentage of DALY rate in 2019. **C** Age-standardized DALY rate attributable to risk factors. DALY, disability-adjusted life-year

In general, smoking was the main contributor to male DALYs of GC in China, Japan, South Korea, Mongolia, East Asia and Pacific, and the world (more than 21%), but the minor contributor to female DALYs (4.2% worldwide) (Fig. 6A). Except for China (about 28% in males and 2.8% in females) and Mongolia (about 21% in males and 2.9% in females), the contribution of smoking to GC showed a downward trend in the world, especially in Japan and South Korea (decline with 9.5 and 6.5% for men, respectively). Although the contribution of smoking was also decreased for female GC in Japan, the corresponding proportion was twice that of China, South Korea and Mongolia. The percentage of GC caused by a high-sodium diet was similarly distributed between men and women, and the contribution kept stable over the three decades, except for a downward trend for female GC burden in Japan and Mongolia. However, the contributions from the high-sodium diet in China, South Korea, and Japan were more than the average level of the world. For all age-specific groups, the contributions from the high-sodium diet were similar to the overall characteristics in 2019 (Fig. 6B). The contributions from smoking were evident among the population aged 40–79 years (Fig. 6B). In the past 30 years, the ASDR of GC attributed to smoking and high-sodium diet in China, Japan, South Korea, Mongolia, East Asia and Pacific, and the world presented a significant downward trend, but the attributable ASDR somewhat stabilized in recent years (Fig. 6C).

## Discussion

The GC burden along with its temporal trends and risk factors in China, Japan, South Korea, and Mongolia from 1990 to 2019, and projections till 2030 were systemically summarized in the current study. Although the ASIR, ASMR, and ASDR of GC continued to decline in most countries, the incident cases, deaths and DALYs of GC in China and Mongolia increased incrementally. It is worth noting that the ASR of GC burden in Japan dropped sharply, resulting in the absolute burden being flat or even decreasing. In addition, the ASR of GC burden increased with age, especially in people aged ≥ 45 years, peaking at the age of 85 in China, Japan, South Korea, and Mongolia. Moreover, the descending trend in ASIR of GC became flattened somewhat in recent years, and the projected ASIR will remain stable in China and elevate slightly in South Korea, partly due to the aging of society and higher incidence among the elderly. Moreover, with the aging and growth of the global population, GC continues to impose a more severe absolute burden on the public health care system of most countries in the world. Recently, Lin and colleagues explored the past temporal trends in the incidence rate of GC from 1988 to 2012 in 43 countries, including China, Japan and South Korea, and further predicted future trends till 2030 using the Bayesian age-period-cohort model, which presented a significant decline in incidence rate in the three countries [25]. However, the older data might limit its projection. For instance, the predicted incidence rate in South Korea would always be higher than that of China, but the actual data were the opposite in recent years in our retrieved data. Yang and colleagues summarized the burden, trends, and risk factors of esophageal cancer in China, Japan and South Korea from 1990 to 2017 based on the GBD 2017 study [26]. Similar design, we compared and further predicted GC burden in China, Japan, South Korea and Mongolia, which would present a comprehensive summarization on upper gastrointestinal tumors in East Asia.

Our results confirmed that the global GC burden in men was more than twice that of women, and the male-to-female ratio was further pronounced in China [1, 27]. However, the reasons for gender disparities in GC burden had not yet been fully elucidated. The contribution of smoking to GC partly explains the higher burden in men, because the age-standardized prevalence of daily smoking worldwide was 25.0% for men and 5.4% for women [28]. Our study verified that the percentage of GC burden due to tobacco was higher in men than in women, especially in China. Other potential risk factors, such as heavy alcohol consumption and low female sex hormones, may increase the risk of GC [29, 30]. These might partly explain the higher burden of GC in men than in women.

The huge geographic inequalities in the ASIR, ASMR and ASDR of GC were observed for both sexes across China, Japan, South Korea, Mongolia, and the world, which may be caused by the variation in the exposure levels of risk factors. Low socioeconomic development is a potential risk factor for the incidence and prognosis of GC [31, 32], which could be epitomized by the SDI in our study. The ASDR of provincial GC in China declined with the increase of SDI, and the annual descending rates in Japan and South Korea were significantly more evident than that of China. The similar ASIR and ASDR of GC, and the relatively slow downward trend in Mongolia were partly restricted by the low socioeconomic development. Moreover, the ASIR in Japan and South Korea were closed to the level of China in 2019, while the ASMR and ASDR of GC in Japan and South Korea were significantly lower than that of China. This may be related to the expanded early screening for upper gastrointestinal cancer, health education promotion, *H. pylori* eradication and control of potential risk factors in Japan and South Korea [33, 34]. For example, South Korea launched a nationwide GC screening plan in 2002, and the Japanese government ratified the insurance coverage of *H. pylori* screening and treatment and post-treatment monitoring of *H. pylori* in 2013 [35].

We further revealed that the risk factors (smoking and the high-sodium diet) presented different attributable patterns for GC development in China, Japan, South Korea, Mongolia, and the world. Tobacco smoking remains the well-known risk factor for GC with a relative risk of around 1.4 times [36–38]. With the implementation and promotion of the WHO Framework Convention on Tobacco Control since 2005, the smoking prevalence rates in most countries and regions have been steadily declining [28, 39], which is the reason for the decline in the contribution of smoking to GC burden during the past 30 years. However, the proportion of CG burden attributable to smoking remained stable in Chinese and Mongolian GC patients, especially in men. Although China has implemented various tobacco control measures and achieved a slight downward trend in smoking prevalence, about 37.5% of male smoking prevalence in 2015 emphasizes strengthening potential control measures [28], such as increasing taxes on tobacco products, prohibiting sales to adolescents, restricting sales and advertising, forbidding smoking in public places, and publicizing the hazard of smoking [40, 41]. The carcinogenic mechanism of smoking on GC is not yet fully understood, but some studies have indicated the chemical carcinogens in smoke may cause genetic damage and epigenetic changes, thereby promoting the development of GC [42, 43].

High salt intake is an important risk factor for GC, which may contribute to gastric carcinogenesis by stimulating the gastric mucosa, inducing intestinal metaplasia, promoting gastric dysplasia [44, 45]. In addition, salted-preserved foods may also contain nitrates to promote the formation of N-nitroso compounds [44, 45]. Our study showed that the proportion of GC-related DALYs caused by high-sodium diets in China, Japan, and South Korea in 2019 exceeded 8.4% except for 7.0% in Japanese women. In addition, except for a significant decline in Japan and Mongolia, the proportion remained stable for the past 30 years in China, South Korea, and the global level. The control of salt intake is an important health measure that involves reducing the incidence of cardiovascular and GC [46, 47], and the healthy strategies in the Japanese diet, traditional Washoku characterized by high consumption of fish and soybean products and low consumption of animal fat and meat, could be are worth learning and recommending [48].

*H. pylori* has been identified as a class I human carcinogen by IRAC, especially for non-cardia GC. In 2015, approximately 4.4 billion people worldwide were positive for *H. pylori* infection [49]. Overall, the prevalence of *H. pylori* has been greatly reduced in the Western countries with around 35%, while the corresponding prevalence remained in developing countries at a high level with more than 50% [49]. The *H. pylori* infection rate in Mongolia is estimated to be 74.1% [50], which is higher than China (55.8%), Japan (51.7%) and South Korea (53.9%) [49]. Robust evidence demonstrates that *H. pylori* eradication can reduce the incidence of GC with the relative risk of 0.54 in the healthy population, and prevent deterioration of mild-to-moderate atrophic gastritis [34, 51], which imply expanded population screening, improving the treatment of *H. pylori* infection, developing effective *H. pylori* vaccines will reduce the rate of death and disability-adjusted life-years of GC [33, 52, 53]. In the current study, the GC burden due to *H. pylori* is not evaluated because it is not included in the GBD database. Catherine and colleagues estimated 0.81 million cancers, mainly non-cardia GC, were caused by *H. pylori* in 2018 [54]. In addition, alcohol consumption, low intake of fruits and vegetables, obesity, poor oral health, low socioeconomic level, Epstein-Barr virus infection, radiation exposure, and family history of GC may increase the incidence of GC [2, 53, 55].

Our research has certain limitations. Firstly, the subtypes of GC, such as cardia GC and non-cardia GC, were not further analyzed due to lack of the necessary data. The epidemiological features and trends of the two subtypes show quite difference. Obesity and gastroesophageal reflux disease usually increase the risk of cardia GC, while *H. pylori* infection, high-salt food diet, and low socioeconomic level are the specific risk factors for non-cardia GC [56]. Moreover, the GC burden attributable to other important risk factors, such as *H. pylori* infection, did not be investigated for the limited information in the GBD database. Finally, the differences in diagnostic methods and consequently accuracy for identifying GC patients among these countries or even subnational regions may affect our results. However, the national cancer registry systems annually collect all cancer burdens with an accurate clinical diagnosis, which somewhat reduces the current concern.

## Conclusions

Although the ASIR, ASMR, and ASDR of GC in most countries continue to decline, the incident cases, deaths and DALYs of GC in China and Mongolia, are on the rise gradually. Notably, the ASR of GC burden in Japan and South Korea with high GC burden has dropped sharply, and their absolute burden has remained flat or even decreased. With the growth and aging of the global population, GC will continue to impose a heavier burden on the public health care system. Therefore, more targeted prevention and treatment strategies, such as *H. pylori* eradication, smoking control, and healthy diet, should be formulated for people adapting to different genders, age groups, and regions. Moreover, plans and measures to improve the early diagnosis rate and new effective treatment methods should be formulated to reduce the burden of GC.

## Supplementary Information

**Additional file 1. Figure S1**. Age-specific rates for incidence, death, and DALY increased with age in China, Japan, South Korea, Mongolia, East Asia and Pacific, and the world in 2019, by sexes. DALY, disability-adjusted life-year. **Figure S2**. The absolute burden of gastric cancer in China, Japan, South Korea, Mongolia, East Asia and Pacific, and the world in 2019, by sexes and age groups. (A) Number of incident cases; (B) Number of incident deaths; (C) Number of DALYs. DALY, disability-adjusted life-year. **Figure S3**. Trends for incidence rate of gastric cancer in China, Japan, South Korea, Mongolia, East Asia and Pacific, and the world from 1990 to 2019 by age groups. (A) Age-specific incidence rate, both; (B) The AAPC in age-specific incidence rate by sexes. AAPC, average annual percentage change. **Figure S4**. Trends for mortality rate of gastric cancer in China, Japan, South Korea, Mongolia, East Asia and Pacific, and the world from 1990 to 2019 by age groups. (A) Age-specific mortality rate, both; (B) The AAPC in age-specific mortality rate by sexes. AAPC, average annual percentage change. **Figure S5**. Trends for DALY rate of gastric cancer in China, Japan, South Korea, Mongolia, East Asia and Pacific, and the world from 1990 to 2019 by age groups. (A) Age-specific DALY rate, both; (B) The AAPC in age-specific DALY rate by sexes. DALY, disability-adjusted life-year, AAPC, average annual percentage change. **Table S1**. DALYs and age-standardized DALY rate from 1990 to 2017 for gastric cancer in the 34 provinces of China.

## Data Availability

The data that support the findings of this study are openly available in Global Health Data Exchange, at http://ghdx.healthdata.org/gbd-results-tool.

## Abbreviations

GC: Gastric cancer
DALYs: Disability-adjusted life-years
ASIR: Age-standardized incidence rate
ASMR: Age-standardized mortality rate
ASDR: Age-standardized DALY rate
AAPC: Average annual percentage change
GBD: Global Burden of Diseases
CI5: Cancer Incidence in Five Continents
SEER: Surveillance, Epidemiology, and End Results
CODEm: Cause of Death Ensemble model
MIR: Mortality-to-morbidity ratio
UI: Uncertainty interval
SAR: Special Administrative Region
SDI: Socio-demographic index
WCRF: World Cancer Research Fund
APC: Annual percentage changes
ASR: Age-standardized rate
CI: Confidence interval
*H. pylori*: *Helicobacter pylori*
BAPC: Bayesian age-period-cohort
RW2: Second-order random walk
