# Supplementary material for: Temporal trend of gastric cancer burden along with its risk factors in China from 1990 to 2019, and projections until 2030: comparison with Japan, South Korea, and Mongolia
Source: Biomark Res. 2021 Nov 16;9:84. doi: 10.1186/s40364-021-00340-6 (PMC8597246; doi:10.1186/s40364-021-00340-6)
Supplement: Supplementary file 1 — Additional file 1. Figure S1. Age-specific rates for incidence, death, and DALY increased with age in China, Japan, South Korea, Mongolia, East Asia and Pacific, and the world in 2019, by sexes. DALY, disability-adjusted life-year. Figure S2. The absolute burden of gastric cancer in China, Japan, South Korea, Mongolia, East Asia and Pacific, and the world in 2019, by sexes and age groups. (A) Number of incident cases; (B) Number of incident deaths; (C) Number of DALYs. DALY, disability-adjusted life-year. Figure S3. Trends for incidence rate of gastric cancer in China, Japan, South Korea, Mongolia, East Asia and Pacific, and the world from 1990 to 2019 by age groups. (A) Age-specific incidence rate, both; (B) The AAPC in age-specific incidence rate by sexes. AAPC, average annual percentage change. Figure S4. Trends for mortality rate of gastric cancer in China, Japan, South Korea, Mongolia, East Asia and Pacific, and the world from 1990 to 2019 by age groups. (A) Age-specific mortality rate, both; (B) The AAPC in age-specific mortality rate by sexes. AAPC, average annual percentage change. Figure S5. Trends for DALY rate of gastric cancer in China, Japan, South Korea, Mongolia, East Asia and Pacific, and the world from 1990 to 2019 by age groups. (A) Age-specific DALY rate, both; (B) The AAPC in age-specific DALY rate by sexes. DALY, disability-adjusted life-year, AAPC, average annual percentage change. Table S1. DALYs and age-standardized DALY rate from 1990 to 2017 for gastric cancer in the 34 provinces of China. [file 40364_2021_340_MOESM1_ESM.pdf]

Temporal trend of gastric cancer burden along with its risk factors in China from 1990 to 2019, and projections until 2030: comparison with Japan, South Korea, and Mongolia

Running title: Comparing gastric cancer in East Asia

5 supplementary figures and 1 supplementary table

Figure S1. Age-specific rates for incidence, death, and DALY increased with age in China, Japan, South Korea, Mongolia, East Asia and Pacific, and the world in 2019, by sexes. DALY, disability-adjusted life-year.

Figure S2. The absolute burden of gastric cancer in China, Japan, South Korea, Mongolia, East Asia and Pacific, and the world in 2019, by sexes and age groups. (A) Number of incident cases; (B) Number of incident deaths; (C) Number of DALYs. DALY, disability-adjusted life-year.

Figure S3. Trends for incidence rate of gastric cancer in China, Japan, South Korea, Mongolia, East Asia and Pacific, and the world from 1990 to 2019 by age groups. (A) Age-specific incidence rate, both; (B) The AAPC in age-specific incidence rate by sexes. AAPC, average annual percentage change.

Figure S4. Trends for mortality rate of gastric cancer in China, Japan, South Korea, Mongolia, East Asia and Pacific, and the world from 1990 to 2019 by age groups. (A) Age-specific mortality rate, both; (B) The AAPC in age-specific mortality rate by sexes. AAPC, average annual percentage change.

Figure S5. Trends for DALY rate of gastric cancer in China, Japan, South Korea, Mongolia, East Asia and Pacific, and the world from 1990 to 2019 by age groups. (A) Age-specific DALY rate, both; (B) The AAPC in age-specific DALY rate by sexes. DALY, disability-adjusted life-year, AAPC, average annual percentage change.

Table S1. DALYs and age-standardized DALY rate from 1990 to 2017 for gastric cancer in the 34 provinces of China

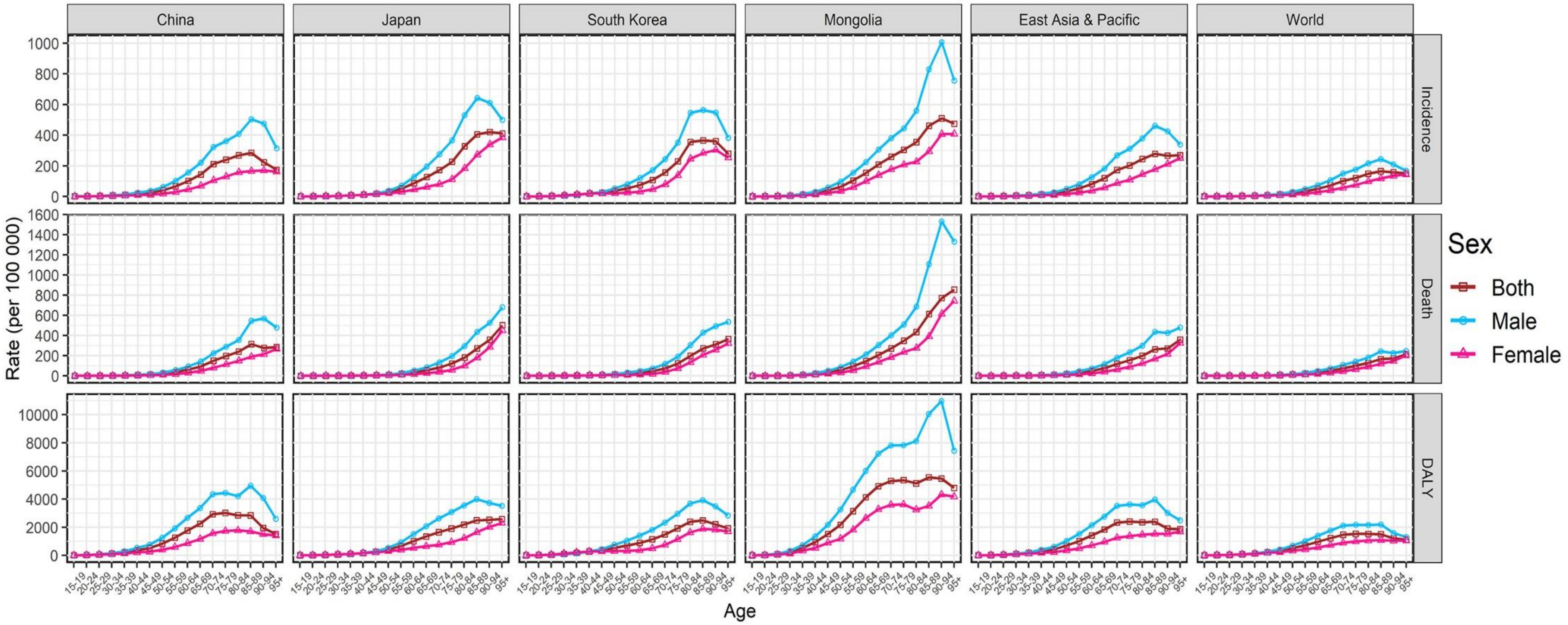

Figure S1. Age-specific rates for incidence, death, and DALY increased with age in China, Japan, South Korea, Mongolia, East Asia and Pacific, and the world in 2019, by sexes. DALY, disability-adjusted life-year.

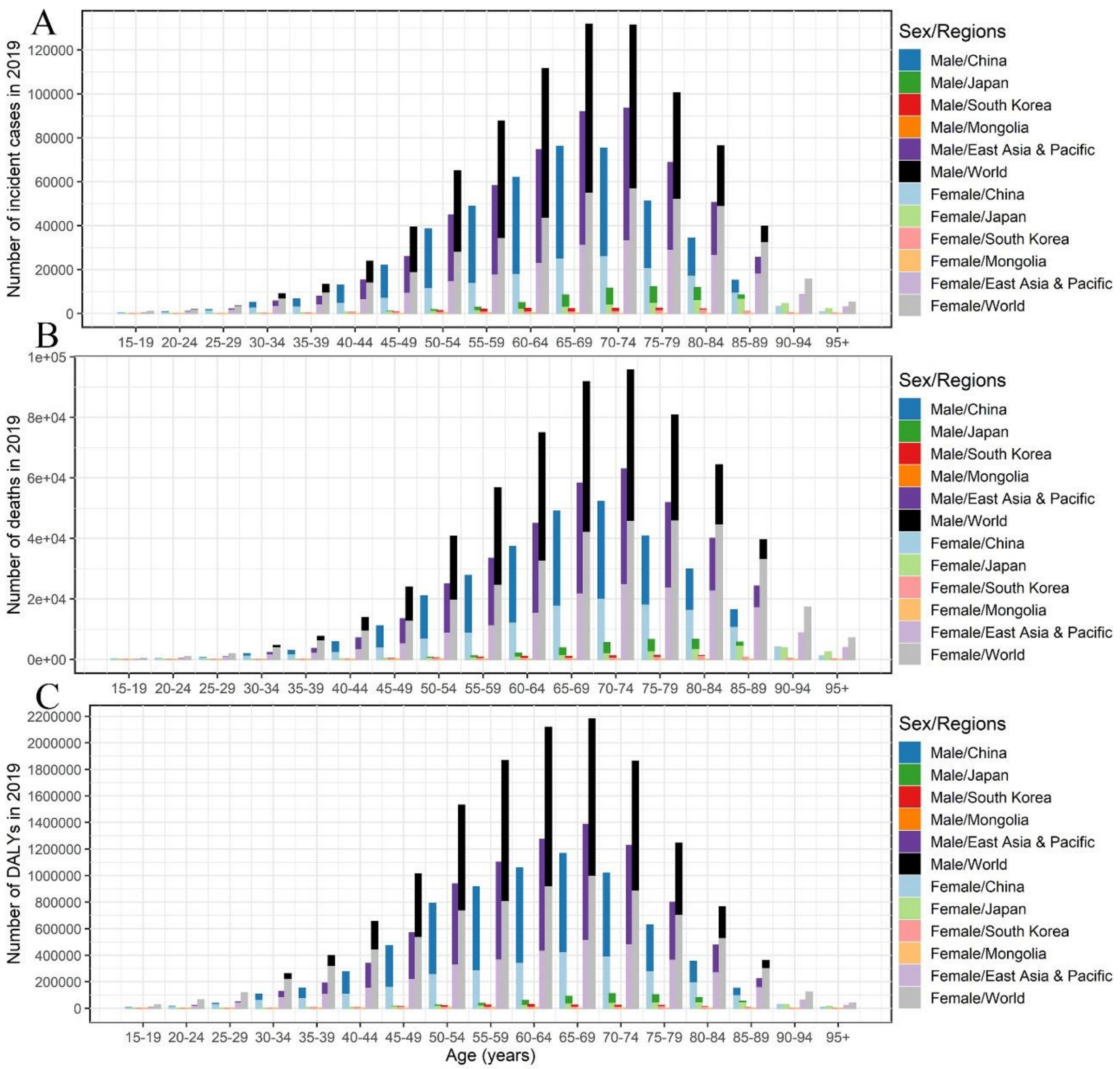

Figure S2. The absolute burden of gastric cancer in China, Japan, South Korea, Mongolia, East Asia and Pacific, and the world in 2019, by sexes and age groups. (A) Number of incident cases; (B) Number of incident deaths; (C) Number of DALYs. DALY, disability-adjusted life-year.

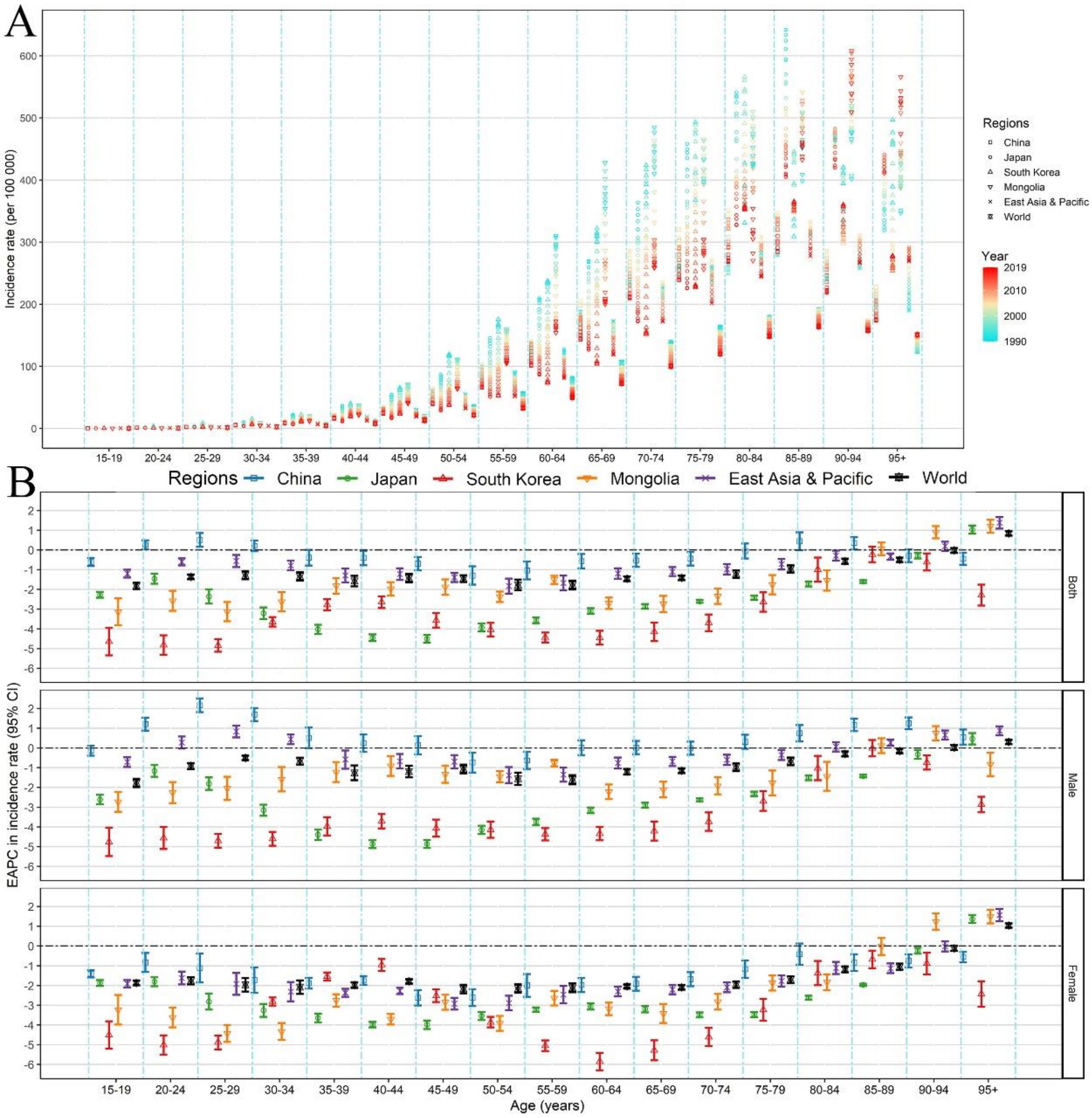

Figure S3. Trends for incidence rate of gastric cancer in China, Japan, South Korea, Mongolia, East Asia and Pacific, and the world from 1990 to 2019 by age groups. (A) Age-specific incidence rate, both; (B) The AAPC in age-specific incidence rate by sexes. AAPC, average annual percentage change.

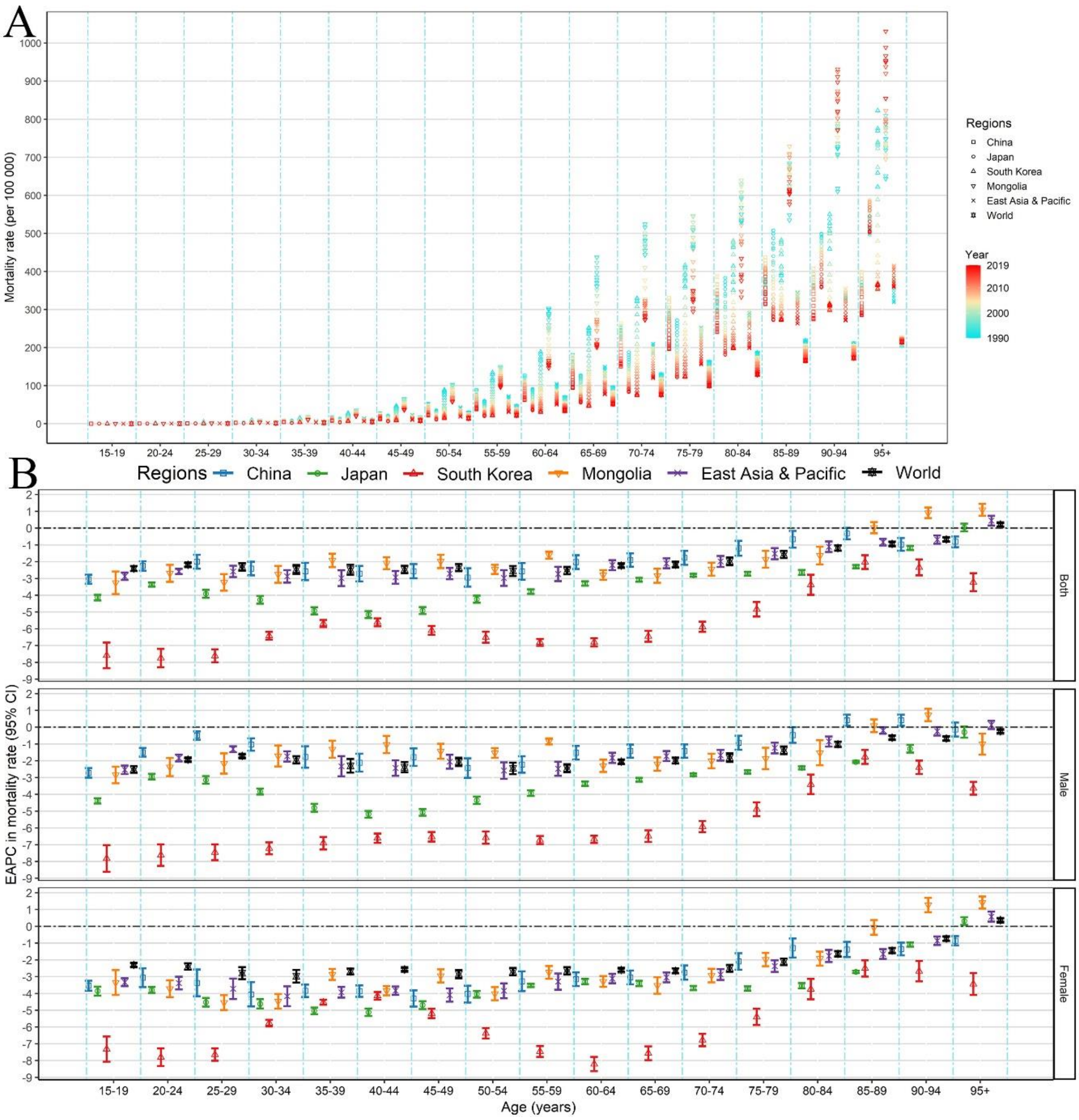

Figure S4. Trends for mortality rate of gastric cancer in China, Japan, South Korea, Mongolia, East Asia and Pacific, and the world from 1990 to 2019 by age groups. (A) Age-specific mortality rate, both; (B) The AAPC in age-specific mortality rate by sexes. AAPC, average annual percentage change.

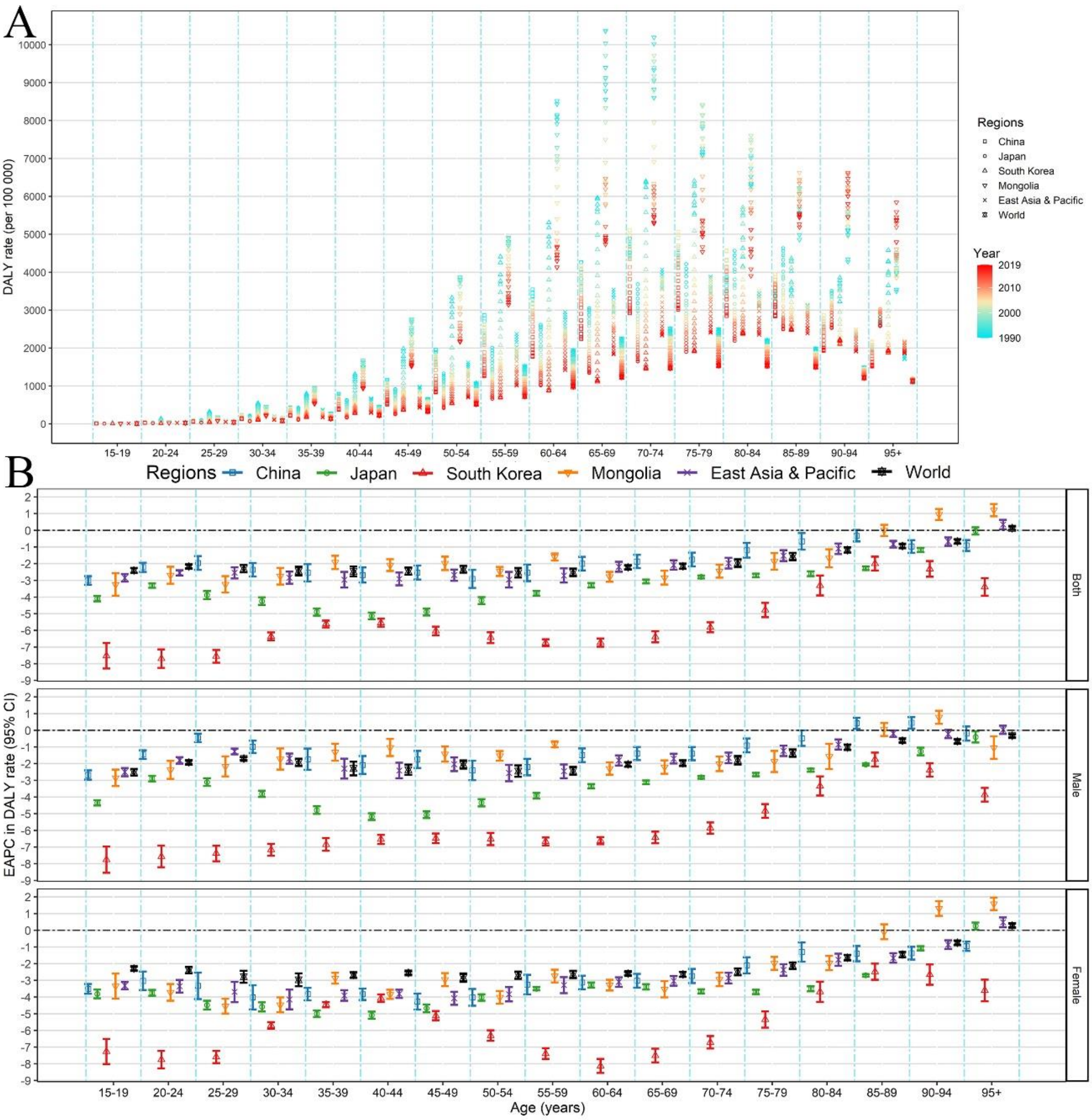

Figure S5. Trends for DALY rate of gastric cancer in China, Japan, South Korea, Mongolia, East Asia and Pacific, and the world from 1990 to 2019 by age groups. (A) Age-specific DALY rate, both; (B) The AAPC in age-specific DALY rate by sexes. DALY, disability-adjusted life-year, AAPC, average annual percentage change.

Table S1. DALYs and age-standardized DALY rate from 1990 to 2017 for gastric cancer in the 34 provinces of China

Location	SDI Quintile	SDI value	Number *10^3 (95% UI) in 1990	Rate/100 000 (95% UI) in 1990	Number *10^3 (95% UI) In 2017	Rate/100 000 (95% UI) in 2017
China	High-middle SDI	0.71	7410 (7140, 7840)	619.45 (596.49, 655)	7810 (7480, 8190)	553.28 (529.25, 579.65)
Beijing	High SDI	0.85	41.0 (35.5, 46.9)	365.6 (316.29, 417.72)	42.3 (34.5, 50.9)	175.5 (142.95, 211.35)
Macau SAR	High SDI	0.83	1.04 (0.953, 1.13)	293.53 (268.65, 317.89)	1.28 (1.07, 1.54)	187.33 (156.5, 224.78)
Guangdong	High-middle SDI	0.76	231 (198, 269)	350.71 (300.65, 408.91)	266 (224, 317)	227.37 (191.95, 271.45)
Tianjin	High-middle SDI	0.80	36.9 (31.9, 41.9)	400.78 (347.31, 455.7)	42.9 (35.9, 51.6)	281 (235.27, 338.12)
Taiwan	High SDI	0.86	63.3 (61.6, 65)	310.19 (301.71, 318.49)	68.3 (64.1, 73)	289.59 (271.6, 309.54)
Yunnan	Middle SDI	0.62	122 (103, 147)	313.18 (264.61, 377.43)	153 (128, 183)	311.61 (260.48, 371.22)
Hong Kong SAR	High SDI	0.82	24.3 (22.1, 26.7)	421.82 (383.82, 464)	24.5 (20.6, 29.2)	325.61 (273.93, 388.3)
Shanghai	High SDI	0.82	102 (88.4, 118)	727.85 (632.55, 842.99)	94 (77.8, 114)	336.6 (278.32, 406.94)
Hainan	Middle SDI	0.68	38.2 (32.5, 44.5)	548.13 (467.12, 638.96)	35.3 (28.5, 43.7)	367.78 (296.94, 455.33)
Chongqing	Middle SDI	0.66	71.7 (61.3, 84.8)	454.43 (388.47, 537.22)	105 (84.5, 129)	391.57 (316.09, 481.04)
Guangxi	Middle SDI	0.68	173 (149, 201)	398.09 (343.66, 462.67)	190 (159, 228)	392.66 (328.94, 470.85)
Jilin	High-middle SDI	0.71	122 (107, 142)	470.78 (412.93, 546.31)	110 (88.9, 131)	394.78 (318.78, 468.22)
Guizhou	Low-middle SDI	0.57	139 (117, 167)	419.57 (353.74, 503.31)	145 (120, 172)	428.89 (353.94, 509.46)
Hunan	Middle SDI	0.68	263 (236, 293)	422.74 (379.44, 471.63)	288 (240, 342)	429.55 (357.69, 509.81)
Tibet	Low-middle SDI	0.47	20 (17.2, 23)	857.15 (738.4, 988.74)	15 (12.4, 17.8)	433.01 (360.14, 515.85)
Inner Mongolia	High-middle SDI	0.71	111 (97, 128)	489.97 (426.61, 564.84)	116 (97.1, 138)	449.14 (374.91, 532.78)
Zhejiang	High-middle SDI	0.74	314 (281, 352)	712.11 (637.01, 797.35)	281 (231, 338)	460.01 (377.17, 552.42)
Heilongjiang	High-middle SDI	0.70	162 (143, 185)	443.54 (390.97, 506.37)	184 (153, 224)	469.63 (388.73, 570.41)
Jiangxi	Middle SDI	0.64	282 (253, 315)	714.13 (640.67, 796.72)	230 (194, 270)	483.29 (408.75, 568.39)
Xinjiang	Middle SDI	0.68	70.6 (62.8, 79.4)	447.4 (397.85, 503.47)	121 (98.7, 149)	495.94 (405.25, 610.87)
Shaanxi	Middle SDI	0.68	115 (103, 130)	334.65 (299.55, 378.34)	214 (172, 263)	542.75 (435.72, 666.41)
Liaoning	High-middle SDI	0.74	211 (182, 242)	517.04 (447.34, 592.47)	254 (209, 304)	569.4 (467.37, 680.2)

Fujian	High-middle SDI	0.71	206 (186, 232)	652.16 (587.18, 733.64)	240 (197, 290)	608.15 (500.67, 734.9)
Hubei	Middle SDI	0.69	280 (252, 308)	503.89 (453.81, 554.59)	358 (302, 426)	647.67 (545.73, 771.01)
Ningxia	Middle SDI	0.64	34.3 (29.4, 39.9)	694.06 (594.71, 808.78)	46.5 (37.5, 57.6)	662.78 (534.43, 820.58)
Sichuan	Middle SDI	0.66	621 (559, 707)	556.18 (500.66, 633.29)	569 (462, 676)	680.97 (553.92, 809.29)
Hebei	High-middle SDI	0.70	415 (373, 459)	652.04 (585.8, 720.46)	529 (433, 630)	691.83 (567.2, 824.77)
Shanxi	Middle SDI	0.70	285 (249, 326)	941.92 (823.16, 1075.2)	269 (213, 329)	695.8 (551.76, 851.53)
Henan	Middle SDI	0.68	703 (643, 769)	796.58 (728.14, 871.23)	679 (571, 796)	705.18 (593.14, 826.79)
Shandong	High-middle SDI	0.74	703 (619, 799)	821.61 (723.56, 933.07)	728 (606, 875)	732.29 (609.28, 880.72)
Jiangsu	High-middle SDI	0.74	621 (562, 691)	901.73 (817.21, 1004.6)	661 (547, 785)	809.5 (669.9, 961.0)
Anhui	Middle SDI	0.62	559 (490, 634)	943.03 (827.3, 1070.1)	517 (434, 614)	845.5 (709.2, 1003.95)
Qinghai	Middle SDI	0.62	42.3 (36.8, 49.8)	893.3 (776.9, 1050.8)	58.1 (46.5, 69.8)	925.2 (739.7, 1110.3)
Gansu	Low-middle SDI	0.60	294 (262, 334)	1242.7 (1107.0, 1410.9)	248 (206, 292)	933.14 (775.1, 1099.4)

DALYs, disability-adjusted life-years; SDI, socio-demographic index; UI, uncertainty intervals, SAR, special administrative region.
